# Apolipoprotein E content of VLDL limits LPL-mediated triglyceride hydrolysis

**DOI:** 10.1016/j.jlr.2021.100157

**Published:** 2021-12-01

**Authors:** Brynne E. Whitacre, Philip Howles, Scott Street, Jamie Morris, Debi Swertfeger, W. Sean Davidson

**Affiliations:** 1 Department of Pathology and Laboratory Medicine, University of Cincinnati, Cincinnati, OH, USA; 2 Department of Pediatrics, Cincinnati Children’s Hospital Medical Center, Cincinnati, OH, USA

**Keywords:** apolipoprotein(s), apolipoprotein E, LPL, lipoprotein metabolism, lipoproteins, VLDL, chylomicrons, metabolic disease, TG-rich lipoproteins, lipolysis, APOC2, apolipoprotein C-II, APOC3, apolipoprotein C-III, APOE, apolipoprotein E, CM, chylomicron, CV, cardiovascular, FA, formic acid, LPDP, lipoprotein-deficient plasma, MW, molecular weight, SDC, sodium deoxycholate, TG, triglyceride, TRL, TG-rich lipoprotein

## Abstract

High levels of circulating triglycerides (TGs), or hypertriglyceridemia, are key components of metabolic diseases, such as type 2 diabetes, metabolic syndrome, and CVD. As TGs are carried by lipoproteins in plasma, hypertriglyceridemia can result from overproduction or lack of clearance of TG-rich lipoproteins (TRLs) such as VLDLs. The primary driver of TRL clearance is TG hydrolysis mediated by LPL. LPL is regulated by numerous TRL protein components, including the cofactor apolipoprotein C-II, but it is not clear how their effects combine to impact TRL hydrolysis across individuals. Using a novel assay designed to mimic human plasma conditions in vitro, we tested the ability of VLDL from 15 normolipidemic donors to act as substrates for human LPL. We found a striking 10-fold difference in hydrolysis rates across individuals when the particles were compared on a protein or a TG basis. While VLDL TG contents moderately correlated with hydrolysis rate, we noticed substantial variations in non-apoB proteins within these particles by MS. The ability of LPL to hydrolyze VLDL TGs did not correlate with apolipoprotein C-II content, but it was strongly inversely correlated with apolipoprotein E (APOE) and, to a lesser extent, apolipoprotein A-II. Addition of exogenous APOE inhibited LPL lipolysis in a dose-dependent manner. The APOE3 and (particularly) APOE4 isoforms were effective at limiting LPL hydrolysis, whereas APOE2 was not. We conclude that APOE on VLDL modulates LPL activity and could be a relevant factor in the pathogenesis of metabolic disease.

Hypertriglyceridemia is defined as a fasting serum triglyceride (TG) level of 150 mg/dl (1.7 mmol/l) or higher (1). Data show that 47% of the US population exhibited fasting hypertriglyceridemia in 2010 (2). While there are genetic mutations that can cause severe familial hypertriglyceridemia or familial combined hyperlipidemia, most patients with hypertriglyceridemia do not have a recognized genetic cause (1). The risk of cardiovascular (CV) events, atherosclerosis, ischemic stroke, and pancreatitis increases with increasing plasma TG concentrations (1, 2). Multiple Mendelian randomization analyses have found a causal link between TG levels and coronary heart disease or CVD events (3, 4, 5, 6). In addition, the Reduction of Cardiovascular Events with Icosapent Ethyl-Intervention Trial found that administration of icosapent ethyl, a prescription omega-3 fatty acid, significantly reduced plasma TGs, CVD events, and CV-related death in patients with elevated TGs, known CVD disease, or were at a risk of developing CVD. Notably, this occurred on top of existing statin therapy (7).

TGs are carried primarily in chylomicrons (CMs) and VLDLs, together referred to as TG-rich lipoproteins (TRLs). Circulating levels of TRL depend on their rate of production in the gut (CM) or liver (VLDL), rate of metabolism by lipases such as LPL in the circulation, and rate of clearance of remnant particles by receptors in the liver (8). During hypertriglyceridemia, postprandial metabolism of TRLs is significantly diminished (9). CMs compete with VLDL for TG hydrolysis by LPL, preferentially hydrolyzing TGs in larger CMs over small CMs (9, 10). In addition, it has been reported that postprandial plasma levels of small CM remnants were directly related to the progression of coronary lesions in postinfarction patients (11). Numerous other groups report the correlation between the rate of postprandial TRL clearance and CV events and/or hyperlipoproteinemia and hypertriglyceridemia (12, 13, 14, 15, 16, 17). Thus, it is important to understand the molecular determinants of TRL clearance in the plasma.

Studies investigating the metabolic fate of VLDL particles demonstrated that their clearance rate is a function of the particle size and lipid and apolipoprotein composition (18). The action of LPL is known to be dramatically stimulated by its cofactor, apolipoprotein C-II (APOC2) (19). Many studies, including in vitro, in vivo, and human genetic studies, have revealed the ability of APOC2 to stimulate LPL activity (20). In addition, lipolysis mediated by LPL is significantly diminished in the absence of APOC2 or with defects in its structure (21). It was found that the APOC2 amino acid residues 44–50, the lipid-binding amino terminus, are critical for the *V*_*max*_ of the LPL hydrolysis reaction (22). In addition, apolipoprotein C-I and apolipoprotein C-III (APOC3) have been demonstrated to inhibit the clearance of plasma TGs, possibly by displacing LPL or APOC2 from lipoproteins or by altering the binding of apoC-containing lipoproteins to their appropriate receptors (20, 23). A host of other proteins including APOE, GPIHBP1, and ANGPTL4 have also been shown to regulate LPL activity. While several studies have investigated the impact of these proteins in various systems including engineered mouse models (24, 25, 26, 27, 28) and in vitro systems using synthetic emulsion substrates, we are aware of no studies that have determined the relative contributions of resident proteins on LPL activity in native VLDL particles isolated from humans. We developed a “plasma-like” in vitro assay for measuring the ability of VLDL particles isolated from different individuals to act as substrates for human recombinant LPL. We then used semiquantitative MS, backed up by specific enzyme-linked immunosorption assays, to correlate activity with particular proteins in VLDL.

## Materials and methods

### Human subjects and plasma collection

The studies reported here utilized two cohorts of nonhospitalized and outwardly normal human subjects who volunteered to come to our laboratory for blood donations (Table 1). The first consisted of nine individuals (five males and four females) whose VLDL was isolated by ultracentrifugation on two visits that were 1 month apart. The second cohort consisted of six individuals (two males and four females, all different from cohort 1) whose VLDL was isolated by size-exclusion chromatography after a single visit. The work was done under an approved Institutional Review Board protocol from the University of Cincinnati and abides by the Declaration of Helsinki principles. All subjects reported that they had no known CVD and were moderate to minimal consumers of alcohol. No subject reported a diagnosis of type 1 or type 2 diabetes or other major metabolic issues; however, one individual in cohort 2 reported a recent diagnosis of maturity-onset diabetes of the young. No subject was on a statin, but many reported common medications, such as multivitamins, oral contraception, and medications for high blood pressure and allergies (Table 1). Overnight fasted plasma was obtained in sodium citrate (BD Vacutainer tubes). For cohort 1, VLDL was immediately (same day as the blood draw) isolated by ultracentrifugation, as detailed later. For cohort 2, aliquots of plasma were frozen immediately at −80°C until thawed for size-exclusion chromatography.

**Table 1:** Characteristics of human volunteer donors for VLDL hydrolysis experiments

Biological Sex Distribution	Age Range (Years)	Body Mass Index	Alcohol Consumption	CVD, Diabetes, Other Chronic Metabolic Disease
	Mean (Range)			
Cohort 1				
Five male, four female	46 (29–66)	23.8 (18.6–30.8)	Moderate/social	None
Cohort 2				
Two male, four female	39 (22–53)	23.9 (21.2–25.7)	Moderate/social	1 (Maturity-onset diabetes of the young)

### Isolation of VLDL and lipoprotein-deficient plasma

For cohort 1, lipoproteins were isolated via density ultracentrifugation as previously described (29). Briefly, ultracentrifugally isolated-VLDL was obtained by sequential ultracentrifugation from fresh plasma by centrifuging in a 70Ti rotor at 360,000 *g* for 18 h to float the VLDL. The bottom fraction was collected, density was adjusted to 1.063 g/ml, and centrifuged for 24 h to float IDL/LDL. The bottom fraction was collected, the density adjusted to 1.21 g/ml, and centrifuged for 48 h. The top fraction, containing HDL, was removed. The remaining bottom fraction containing the lipoprotein-deficient plasma (LPDP) was collected and dialyzed into PBS (10 mM PBS, 140 mM NaCl, 0.01% EDTA, 0.01% azide [pH 7.4]). This fraction contained most soluble and nonlipid-associated proteins in plasma. A single batch of LPDP was used as the basal media for all LPL activity assays reported here.

### LPL assay by size-exclusion chromatography

Many LPL assays label substrate lipoproteins with radiolabeled or fluorescent TG substrates in order to track TG hydrolysis. To keep our lipoproteins as native as possible, we elected to measure endogenous TG mass enzymatically using colorimetric kits. Since these assays report on free glycerol generated after complete lipolysis of intact TG molecules, we needed to develop assay systems that separated intact TG in lipoproteins from any free lysolipid or glycerol generated by LPL. In our initial studies, we did this by subjecting the LPL reaction mixture to size-exclusion chromatography after it was stopped by the addition of the lipase inhibitor Orlistat. In vitro LPL assays were performed by adding isolated VLDL (20 mg/dl) and proteins of interest to LPDP in volumes ranging from 50 to 350 μl of LPDP or whole plasma depending on the experiment performed (see size-exclusion desalting plate vs. fast protein liquid chromatography methods described later). Lipoproteins and any added apolipoproteins were incubated at 37°C for 1 h in a shaker at 150 rpm. Recombinant human LPL (R&D Systems; catalog number: 9888-LL-100), stored at −80°C in aliquots of 0.0321 μg/μl until use, was added to each tube (0.131–0.525 μg depending on the experiment) and incubated with the lipoproteins for 30–60 min at 37°C on a shaker. The reaction was quenched with 50 μM (final concentration) of Orlistat stored as a 2 mM stock (Sigma-Aldrich; catalog number: O4139). The samples were then run via fast protein liquid chromatography over a single Superose 6 size exclusion column (10/300 GL; GE Healthcare Lifesciences, Pittsburgh, PA). The resulting fractions were assayed for both TG and phospholipid by enzymatic kit as described previously. The results showed ample separation of intact VLDL (and its remaining TG) and the free glycerol generated during the LPL reaction, which were found near the total volume of the elution. The area under the curve for the VLDL fractions was then summed. Total VLDL TG hydrolysis was determined by subtracting the TG value of VLDL with LPL from an identical sample that lacked LPL. This difference is defined for the purposes of this study as percent of TG hydrolysis. We noted minimal hydrolysis in the absence of exogenous LPL indicating little contribution of any endogenous LPL in the plasma sample. Orlistat, present during the entire experiment, was included as a negative control to assure that all effects were due to the addition of the exogenous lipase.

### Higher throughput LPL assay by 96-well desalting plate

Given the time-intensive nature of size-exclusion separations described previously, we developed a more rapid assay that used a 96-well desalting plate to separate intact TG from glycerol. About 10 μg of VLDL protein was added to 41–46 μl of LPDP. The reaction was initiated by adding 4 μl of human recombinant LPL from a stock solution of 0.0321 μg/μl in storage buffer, giving a final reaction volume of 50 μl. After incubation at 37°C for 30 min with moderate shaking, the reaction mixture was applied to a Zeba Spin Desalting Plate (Thermo Scientific; size-exclusion chromatography/proprietary resin), which was prewashed in PBS buffer. The plate was centrifuged at 1,000 *g* for 1 min. This fraction was designated the “flow-through.” The ∼550 μl resin columns were then eluted with successive additions of 30 μl followed by a 1,000 *g* for 1 min spin. Careful analysis showed that intact VLDL particles eluted entirely in elution fractions 1 and 2, whereas free glycerol eluted in fraction 4. Fractions 1 and 2 were pooled and assayed for protein and TG. Like for the size-exclusion chromatography method, percent of hydrolysis was calculated by subtracting intact TG from samples that included LPL from those that lacked it and dividing by the starting TG level. Similar controls as described previously were included, that is, Orlistat. Human plasma APOE (Sigma-Aldrich; SRP6303; stored at −80°C until use and reconstituted as 1 μg/μl) and human recombinant APOE2-4 (Sigma-Aldrich; SRP4760, SRP4696, AK3234; stored at −20°C until use and reconstituted as 1 μg/μl) were purchased for in vitro APOE addition experiments. APOE was incubated with VLDL in LPDP for 1 h at 37°C using between 0.5 and 5 μl depending on the assay.

### Mass Spectrometry

Sodium deoxycholate (SDC), DTT, iodoacetamide, formic acid (FA), acetic acid, acetonitrile, methanol, and ammonium bicarbonate were purchased from Sigma-Aldrich (St. Louis, MO). Proteomics sequencing-grade modified trypsin was purchased from Promega (Madison, WI).

Samples undergoing MS analysis were dialyzed into 50 mM ammonium bicarbonate (pH 8.1), and 50 μg of protein determined by modified Lowry assay (30) from each donor were used for preparation. Preparation methods were modified from previously described methods (31, 32). Briefly, the samples were mixed with 3% SDC and 500 mM DTT for 30 min at 60°C. The samples were then alkylated with 1 M iodoacetamide for 60 min at room temperature and protected from light. The samples were then diluted to an SDC concentration of 1% and digested overnight with 0.2 mg/ml trypsin. The following morning, 10% FA was added to precipitate the SDC (5% of final volume of sample). The samples were then centrifuged at 15,000 *g* at 4°C for 15 min. The supernatants were carefully removed and lyophilized to dryness by SpeedVac and stored at −20°C until MS analysis.

LC-MS/MS analyses were performed on an Agilent 6550 iFunnel Q-TOF LC/MS. Dried samples were reconstituted in FA/water (0.1/99.9, v/v), and 5 μl (∼17 ug protein) was injected. Peptides were eluted at 100 μl/min using a varying mobile phase gradient from 95% phase A (FA/water, 0.1/99.9, v/v) to 32% phase B (FA/acetonitrile, 0.1/99.9, v/v) for 60 min (0.45% per min), then from 32% B to 90% B in 13 min with re-equilibration. The instrument was operated in positive ion mode for 84 min, where each cycle consisted of one TOF-MS scan (0.200 ms accumulation time, in a *m/z* 300–1,400 window) followed by 20 information-dependent acquisition mode MS/MS scans on the most intense candidate ions selected from the initially performed TOF-MS scan during each cycle. A blank was run between each sample.

Peptide spectral data were searched against the UniProtKB/Swiss-Prot Protein knowledgebase (released March 2021; 565,254 sequences) for *Homo sapiens* (20,600 sequences) using Mascot through Matrix Science. Data were constrained to tryptic digestion with a maximum of three missed cleavages. Carbamidomethylation was set as a fixed modification and Met oxidation as a variable modification. Peptide and MS/MS mass tolerance was ±0.15 Da. Scaffold (version 4.3.4) was used for MS/MS-based peptide validation using X! Tandem (2010.12.01.1). Proteins and peptides were constrained to >99.9% and 95% identification probability, respectively. In addition, proteins were only accepted if they contained a minimum of three unique peptides. Raw spectral counts were normalized to the apoB spectral counts found within each respective sample to adjust for small differences in protein mass injected onto the mass spectrometer. In certain analyses, peptide spectral data were searched again against the UniProtKB/Swiss-Prot Protein knowledgebase using Maxquant (version 1.6.5.0). Data constraints, modifications, and others remained consistent as described previously. To assess the intra-assay and interassay variability of the LC/MS/MS method, a standard of apolipoprotein A-I was run with each preparation.

### Gel electrophoresis

Samples were dissolved in gel loading buffer containing 4% SDS and 10 mM DTT and 0.6 mg Coomassie blue. Samples were separated using SDS-PAGE in parallel lanes on 4–15% separation gel (thickness of 1 mm, containing 15 wells). After electrophoresis, gels were fixed in a fixing solution (10% acetic acid), and protein bands were then visualized using Coomassie G-250 staining.

### Statistical analysis

Graphics were generated with GraphPad Prism software, version 9.0 (GraphPad) or SigmaPlot, version 11.2 (Systat Software Inc.). Statistical analyses were performed in SigmaPlot, version 11.2. Most analyses used a one-way ANOVA using a Shapiro-Wilk normality test and an equal variance test. If statistical differences were detected (*P* < 0.001), a Tukey honest significant difference test was performed with a threshold of *P* < 0.05.

## Results

Our goal was to create a TG hydrolysis experimental system that *i*) reproduced plasma conditions as closely as possible, *ii*) allowed for the manipulation of reaction components to test their effect on LPL activity, and *iii*) had enough throughput to allow a wide range of sample comparisons with rigorous statistics. We elected to avoid reconstituted substrates such as TG emulsions or reassembled lipoproteins containing labeled lipids and targeted physiologically assembled VLDL particles using straightforward TG mass colorimetric assays. The use of a glycerol-based assay presented a technical hurdle, however. These assays work by first digesting TG with exogenous lipases to liberate the free glycerol backbone, which is oxidized for eventual reaction with a color producing probe. To measure LPL activity, we needed to measure remaining intact TG in VLDL in the absence of any free glycerol generated by LPL as well as the significant amount of glycerol present in the buffer of our commercially purchased LPL preparations. In initial experiments, we elected to separate the reaction components by size-exclusion chromatography.

**Fig. 1:**
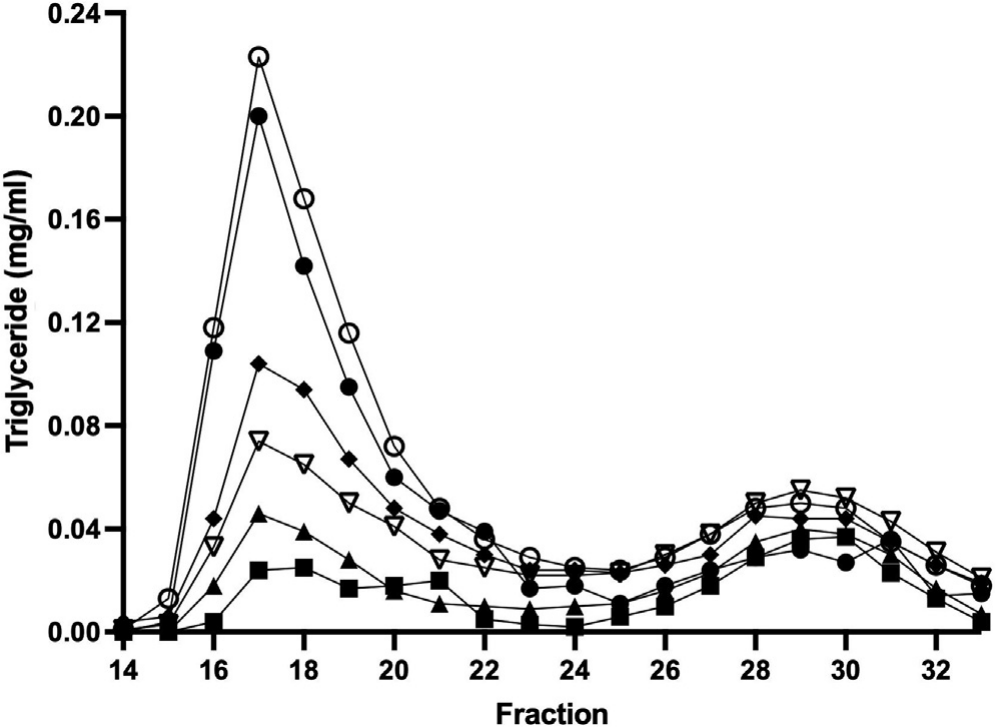
LPL-mediated TG hydrolysis in VLDL fractions in whole plasma separated by size-exclusion chromatography. Citrated plasma (350 μl) was incubated with increasing amounts of recombinant human LPL in vitro and then separated by fast protein liquid chromatography (FPLC) over a Superose 6 gel filtration column in PBS as described in the Materials and methods section. About 300 μl fractions were collected, and TG was quantified colorimetrically. VLDL eluted within fractions 15–23 and LDL eluted in fractions 25–33 in this system. Orlistat, a lipase inhibitor, was used as a control and to quench the reactions. Samples include whole plasma without exogenous LPL (closed circle), whole plasma with 1.5 μg/ml LPL and 50 μM Orlistat (open circle), plasma with 0.37 μg/ml LPL (closed diamond), plasma with 0.75 μg/ml LPL (open downward triangle), plasma with 1.0 μg/ml LPL (closed upward triangle), and plasma with 1.5 μg/ml LPL (closed square). Experiments were performed in triplicate with representative traces shown.

We first tested the ability of human recombinant LPL to hydrolyze TGs in whole human plasma in vitro. Plasma was incubated with varying amounts of human recombinant LPL for 1 h. The reaction was quenched using Orlistat, a lipase inhibitor, and each sample was separated by size exclusion on a single Superdex column as described in the Materials and methods section. Each fraction was assayed for intact TG by colorimetric assay. Figure 1 shows that human plasma contained TG in two peaks centered at fractions 17 (peak 1) and 29 (peak 2). Peak 1 contained VLDL-sized particles, and peak 2 contained LDL-sized particles (note: HDL-sized particles also contain small amounts of TG, but those fractions were not included in this experiment). When 0.131 μg LPL (0.37 μg/ml) was added to plasma, the TG content of VLDL was reduced by about 50% as measured by the decreased area of peak 1. LDL TG content was affected to a lesser extent indicating a preference of LPL for VLDL. As the concentration of LPL was systematically increased, VLDL TG content was reduced to near baseline. When the lipase inhibitor Orlistat was added to the reaction prior to introduction of the highest amount of LPL, VLDL TG content was slightly higher than plasma alone indicating the effectiveness of the inhibitor and suggesting that a small amount of endogenous lipase activity was present in the plasma.

**Fig. 2:**
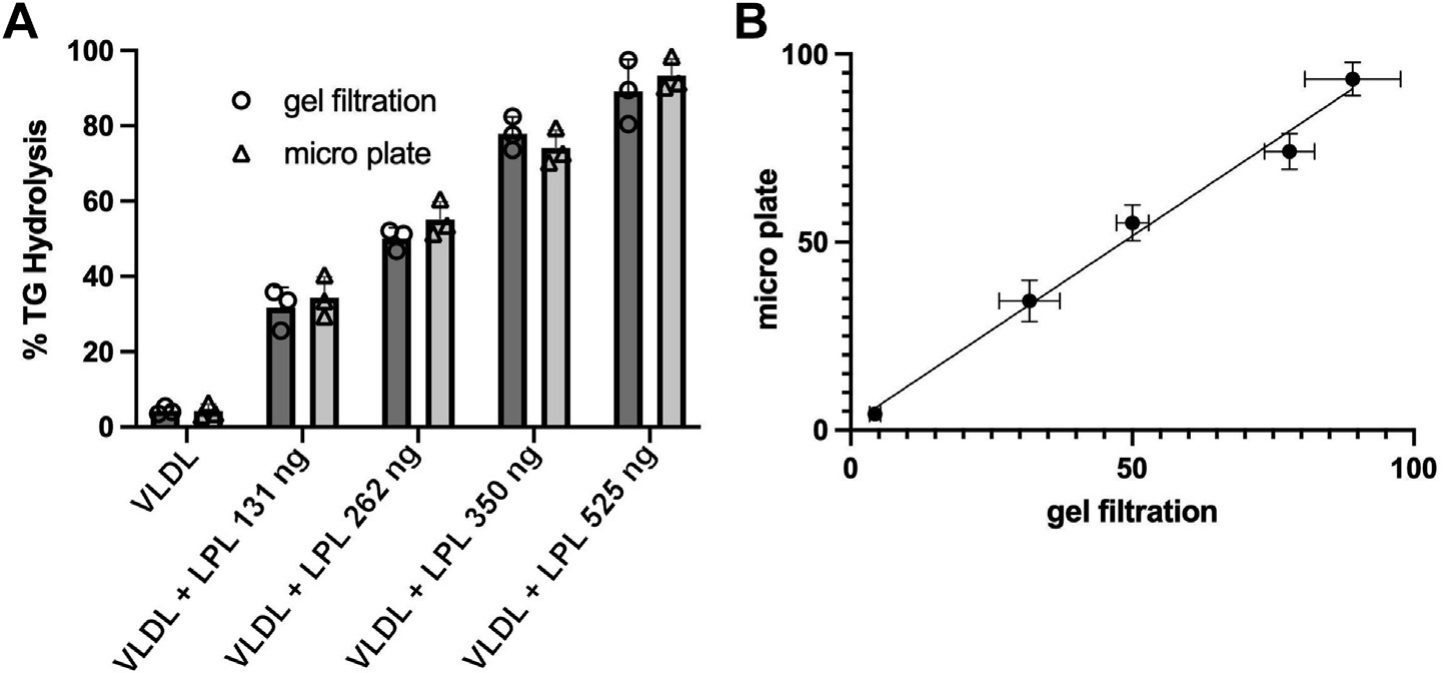
Comparison of rapid microplate assay to size-exclusion chromatography assay of LPL activity. Human VLDL isolated by ultracentrifugation from a single donor at 20 mg/dl (protein) in LPDP was incubated with increasing amounts of human LPL for 30 min at 37°C. The samples were then analyzed by size-exclusion chromatography on a Superose 6 column (as for Fig. 1) or put through the 96-well desalting plate protocol described in the Materials and methods section. A: Percent of TG hydrolysis by LPL as analyzed by both methods. B: Correlation plot of TG hydrolysis as measured by the microplate and size-exclusion methods. The line shows a simple linear regression (*r*^2^ = 0.9708). All experiments were performed in triplicate, and error bars indicate one sample standard deviation.

The size-exclusion chromatography method depicted in Fig. 1, while effective, is labor intensive and not amenable to high sample throughput. Furthermore, we desired a more flexible system where lipoproteins and apolipoproteins could be added in different combinations to measure the effect on LPL hydrolysis under plasma-like conditions. We elected to use LPDP as the base solution for our assay. This maintains the physiological pH buffering systems and oncotic/charge environment from high abundance circulating proteins like albumin and others. This allowed the controlled addition of isolated reaction components such as VLDL from various donors, individual apolipoproteins, LPL, and eventually other mediating components such as HDL. We also miniaturized the lipolysis reaction so that it could be accomplished on 96-well filter plates, allowing separation of the problematic glycerol from the lipoprotein substrates. To validate the method, we exposed ultracentrifugally-isolated VLDL from multiple human donors to human recombinant LPL in LPDP and compared the results to similar experiments carried out with the full-scale size-exclusion chromatography method used in Fig. 1. Figure 2A shows the comparison of the size-exclusion method (open circles) and microplate method (open triangles) for VLDL as monitored by total percent TG hydrolysis (see Materials and methods section for how this was calculated). The VLDL particles without LPL exposure underwent minimal TG hydrolysis, which increased with increasing amounts of LPL for both methods. Figure 2B shows a correlation plot for both methods. A linear regression analysis confirmed concordance between the methods with an *r*^2^ of 0.9708. We used the microplate method for all subsequent analyses in this study.

**Fig. 3:**
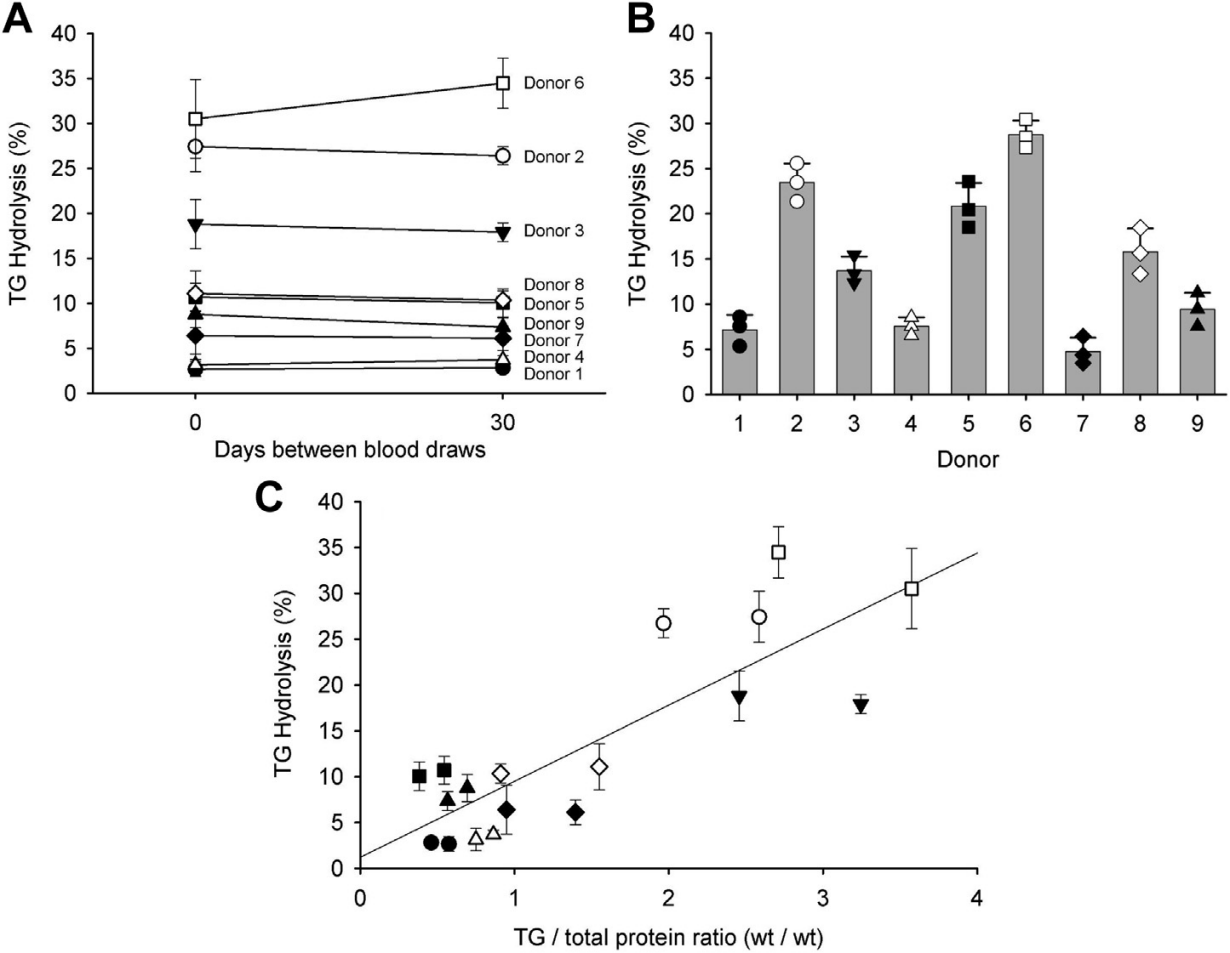
Variation in LPL hydrolysis of ultracentrifugally isolated VLDL from nine donors in cohort 1. A: VLDL was isolated by ultracentrifugation from nine apparently healthy volunteers at *T* = 0 days, then again at *T* = 30 days. Compared at equal TG mass (20 mg/dl), the VLDL particles from each donor were evaluated as substrates for a fixed amount (0.262 μg) of human LPL. The percent of TG hydrolysis is plotted for each donor at each time point. B: We also compared the VLDL samples at equal protein concentrations (20 mg/dl). Data are shown from the *T* = 0 point. A one-way ANOVA indicated significant differences exist among the samples (*P* < 0.001). C: Correlation between TG/protein ratio of each VLDL particle and LPL hydrolysis. The line shows a simple linear regression through all data shown in panel (A), *r*^2^ = 0.6906. For all panels, experiments were performed in triplicate, and error bars show one sample standard deviation. For all panels, filled circle, donor 1; open circle, donor 2; filled down triangle, donor 3; open up triangle, donor 4; filled square, donor 5; open square, donor 6; closed diamond, donor 7; open diamond, donor 8; and filled up triangle, donor 9.

To evaluate the inherent variability of VLDL from different individuals to act as a substrate for LPL, we isolated VLDL from nine normolipidemic human plasma donors (cohort 1; Table 1). The lipid contents of the isolated VLDL particle, with respect to protein content, are shown in Table 2. To assess the temporal stability of any differences noted, we performed identical isolations 1 month later using the same donors. Figure 3A shows the percent TG hydrolysis of each donor's VLDL particles compared on an equal TG basis. We were struck by the wide range of values between the subjects. Particles from donors 1, 4, 7, and 9 exhibited relatively low percent TG hydrolysis as compared with donors 2, 3, and 6 with the others falling in between. This variance was observed when the VLDL samples were compared at either equal TG content or equal protein (Fig. 3A vs. Fig. 3B). This variability was also apparent when the LPL assays were carried out in plasma. The size exclusion profiles of each plasma sample prior to and after addition of LPL are shown in supplemental Fig. S1. Since previous reports have indicated that LPL hydrolysis depended heavily on the TG content of the substrate particles, we plotted the TG to protein weight ratio of the particles versus the TG hydrolysis rate (Fig. 3C). We noted a moderate positive correlation that was particularly affected by the particles with the highest TG/protein ratios. However, since the TG/protein ratio varied within individuals at the different time points, but the hydrolysis rate was remarkably consistent over that time frame, we looked for other factors responsible for the variability in hydrolysis.

**Table 2:** Characteristics of ultracentrifugally isolated VLDL particles from the subjects of cohort 1

Donor	TG/Protein Ratio (wt/wt)	Phospholipid/Protein Ratio (wt/wt)	Total Cholesterol/Protein Ratio (wt/wt)
1	8.90	0.93	1.35
2	9.11	0.89	0.80
3	11.7	0.99	1.33
4	8.39	1.07	2.20
5	11.5	0.98	1.30
6	11.3	0.97	1.53
7	10.6	0.94	1.12
8	11.5	0.92	1.08
9	8.90	0.97	1.24
Average ± SD	10.15 ± 1.43	0.96 ± 0.05	1.33 + 0.39

**Fig. 4:**
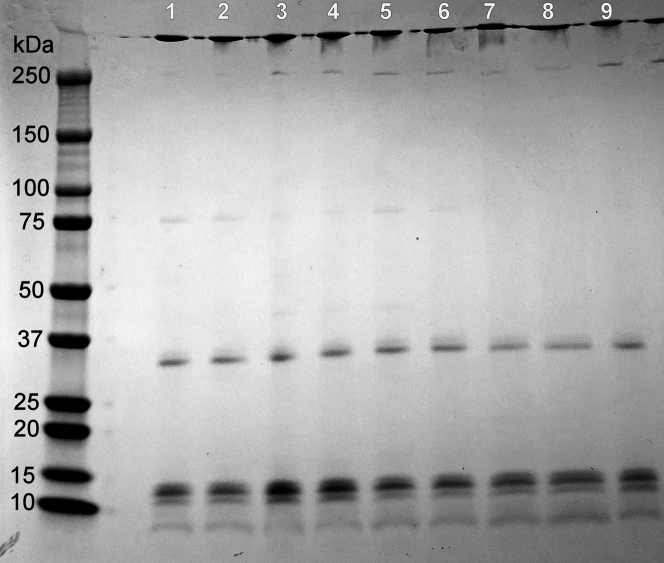
SDS gel electrophoresis of VLDL particles isolated from cohort 1. Ultracentrifugally isolated VLDL obtained from each donor in Fig. 3 was loaded by equal protein on a 4–15% SDS gradient gel and run at 200 V for 30 min under reducing conditions followed by Coomassie staining. The lane numbers correspond to the donor who provided the particles. Expected protein molecular weights for common VLDL: apoB100 (MW, 480 kDa), APOE (MW, 34 kDa), and the apoCs (MW, between 6.6 and 8.8).

**Fig. 5:**
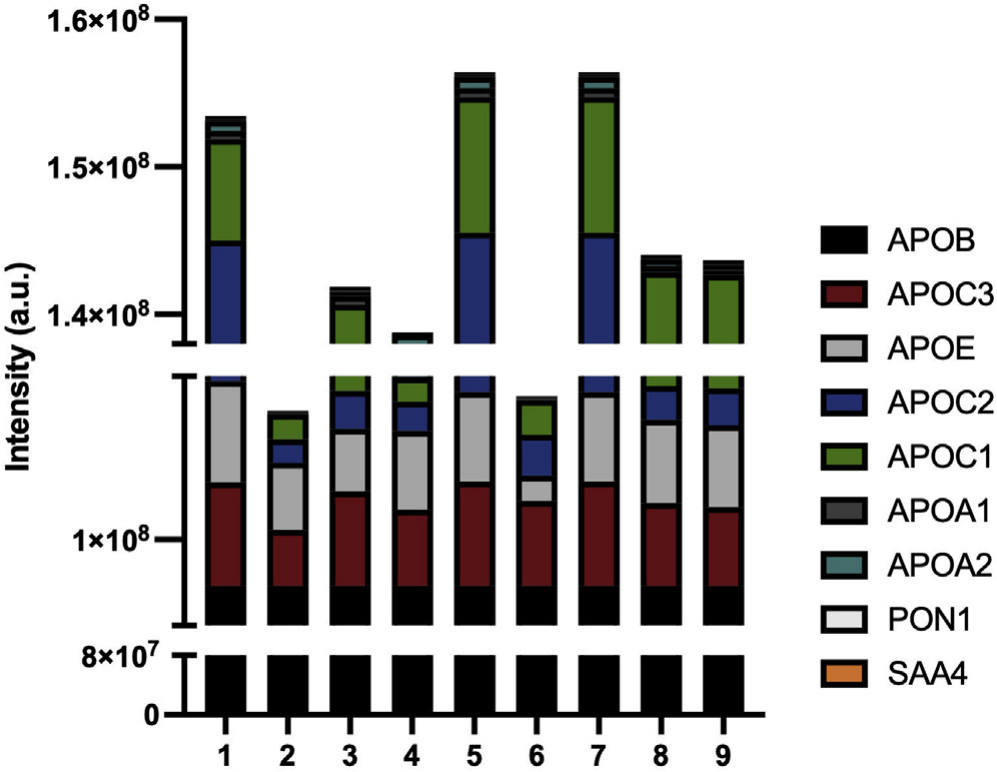
MS-based proteomic analysis of ultracentrifugally isolated VLDL from each donor in cohort 1. About 50 μg of total VLDL protein from each donor was analyzed by MS after delipidation, reduction, alkylation, and digestion with trypsin as described in the Materials and methods section. Data are expressed as the label-free quantitation intensity as calculated by MaxQuant. The plotted data are an average of three separate runs and normalized to apoB concentration.

Each individual’s VLDL particles were separated by denaturing PAGE and visualized by Coomassie blue staining (Fig. 4, loaded at equal protein masses). Overall, the protein banding pattern was similar across the samples with all particles containing APOB100 (molecular weight [MW] of 480 kDa) as a major band as well as APOE (MW of 34 kDa) and the apoCs (MW between 6.6 and 8.8) as expected. However, there were additional bands in some samples, and the intensities of the various bands varied across the particles from each subject. To better assess these differences, an MS analysis was performed on each donor’s VLDL as described in the Materials and methods section (Fig. 5). For each analysis, samples were prepared based on equal total protein content, then the spectral counts for APOB were normalized across samples for comparison. In agreement with the SDS gel analysis, VLDL particles from each donor contained the same most abundant proteins: APOB, APOE, and the APOC family. However, the quantity of several protein constituents varied significantly between individuals. In addition, the proteome remained relatively consistent within individuals between the two time points evaluated (supplemental Fig. S2), unlike the TG contents noted previously. This suggested to us that a component of the proteome may be responsible for much of the observed variability in LPL hydrolysis.

**Fig. 6:**
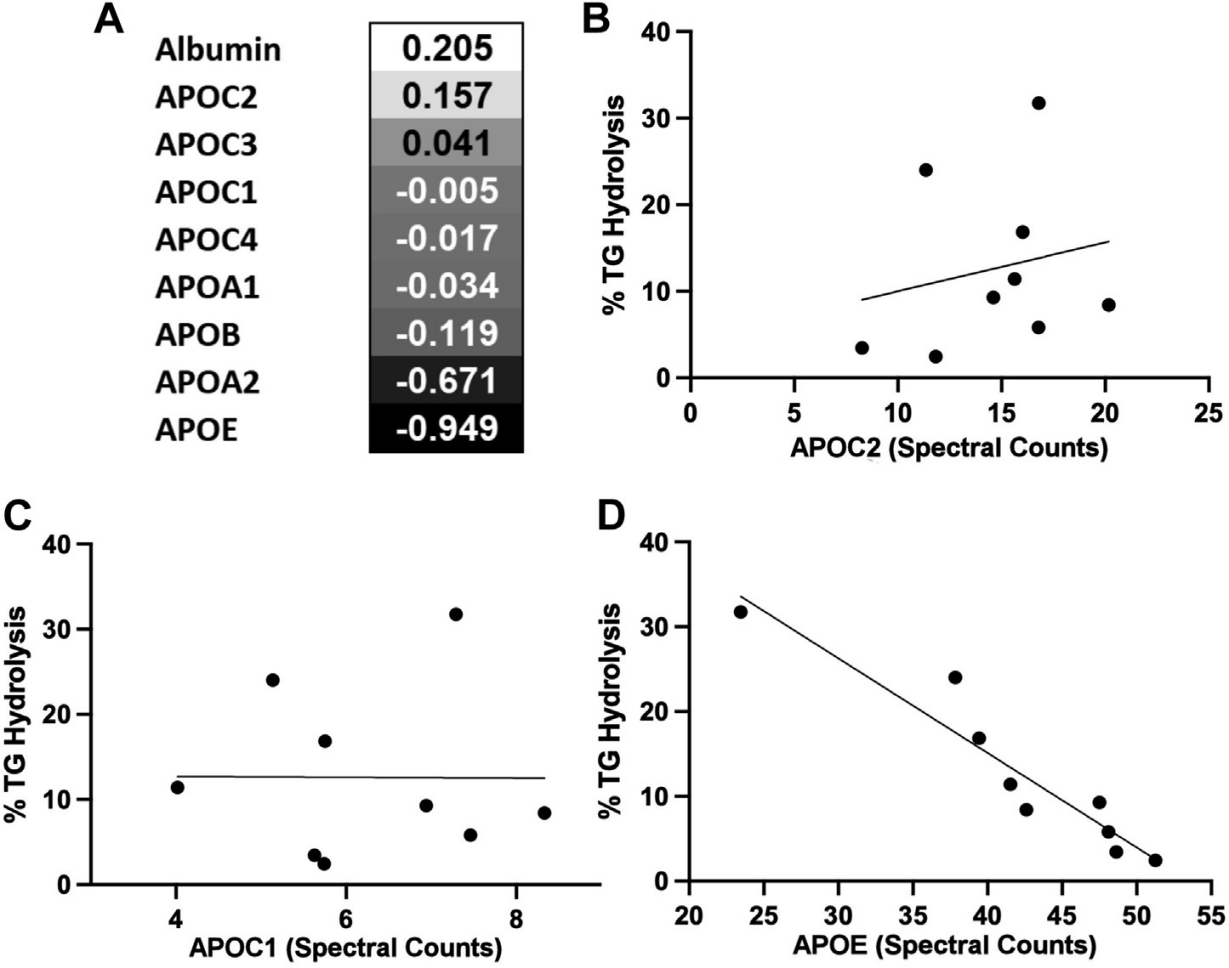
Correlation of VLDL proteome abundance and TG hydrolysis by LPL among donors. A: A Pearson correlation analysis was performed between the percent of TG hydrolysis in the presence of human LPL and the MS spectral counts for each detectable VLDL proteome member. The correlation coefficients are listed for the top nine most abundant proteins detected. B: Correlation between percent of TG hydrolysis and APOC2 spectral count. The line shows a simple linear regression (*r*^2^ = 0.157). C: Correlation between percent of TG hydrolysis and APOC1 spectral count. The line shows a simple linear regression (*r*^2^ = 0.005). D: Correlation between percent of TG hydrolysis and APOE spectral count. The line shows a simple linear regression (*r*^2^ = 0.949). The data are an average of three separate runs and normalized to apoB concentration from the *T* = 0 time point.

We performed a correlation analysis that compared the total percent TG hydrolysis in VLDL to the spectral counts for each protein found by MS (Fig. 6). We found no correlation between APOC2 abundance and LPL's ability to hydrolyze TGs (*r* = 0.1570; *r*^2^ = 0.0418 by linear regression), nor was there an inverse association for APOC3, APOC1, or APOC4. However, there was a striking negative correlation between APOE abundance and LPL hydrolysis (*r* = −0.950; *r*^2^ = 0.9025). Interestingly, the presence of APOA2 demonstrated a moderate negative correlation with LPL-mediated TG hydrolysis also.

**Fig. 7:**
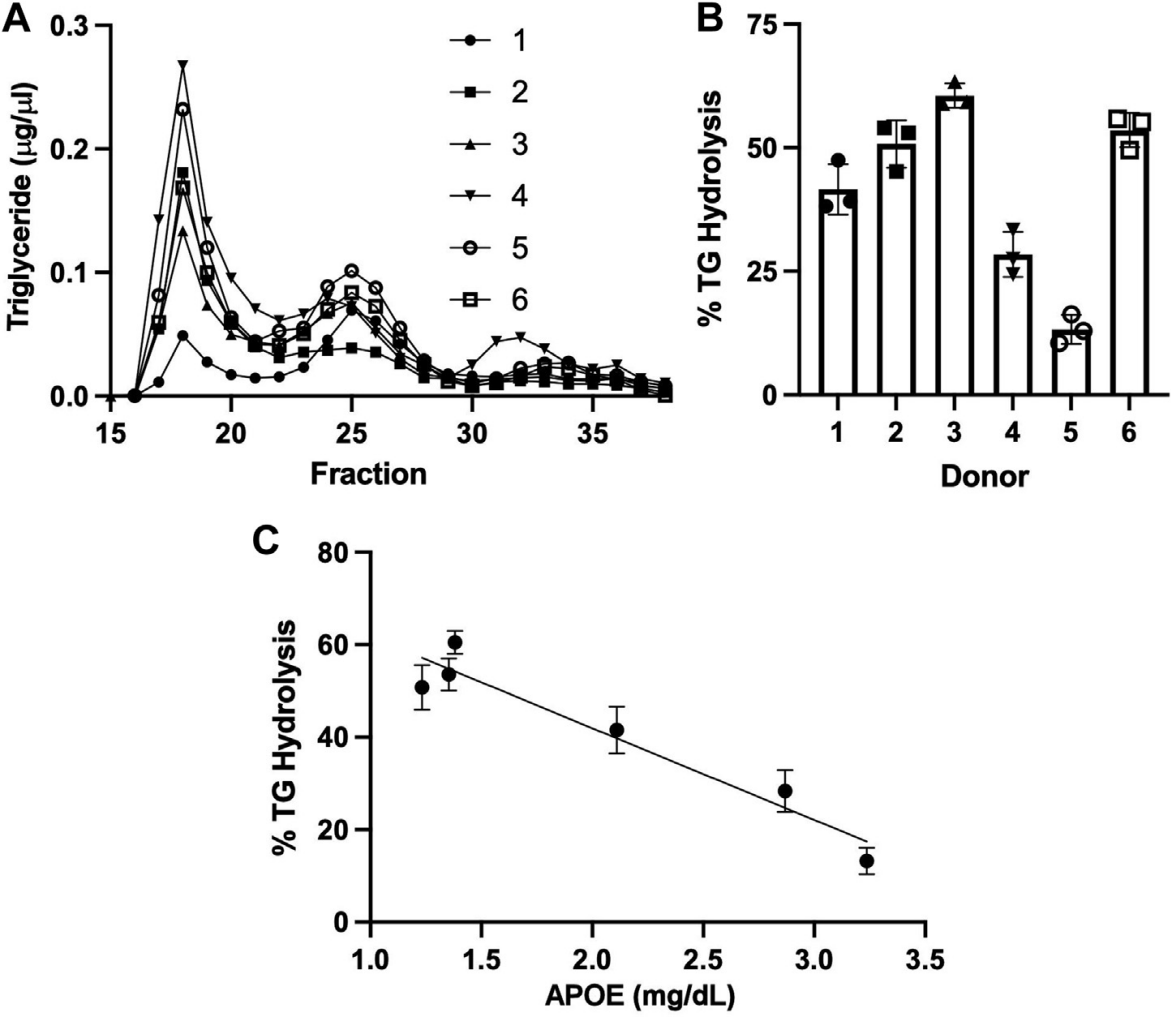
Validation of the APOE effect on LPL-mediated VLDL TG hydrolysis in a second cohort of subjects. A: A second group of six subjects was recruited (cohort 2). Instead of isolating VLDL by ultracentrifugation as in cohort 1, each donor plasma was separated by size-exclusion chromatography. The traces, as tracked by TG, are shown for each donor (see the legend). In this system, VLDL elutes between fractions 15 and 20, LDL is centered on fraction 25, and HDL elutes after about fraction 32. B: TG hydrolysis of pooled VLDL fractions assayed at equal TG concentration as in Fig. 3 (n = 3, error bars show one sample standard deviation). A one-way ANOVA indicated that significant differences existed between the samples (*P* < 0.001). C: Correlation of TG hydrolysis versus concentration of APOE measured by ELISA and normalized to APOB content (also by ELISA). Line shows a simple linear regression (*r*^2^ = 0.889).

To confirm the strong correlation between APOE content and TG hydrolysis by LPL, we engaged a second cohort of human donors. In this experiment, we elected to isolate VLDL using size-exclusion chromatography to rule out possible alterations to the particle proteome that may have occurred because of the high salt conditions employed for density ultracentrifugation isolation in cohort 1. In addition, we used an ELISA assay to more rigorously quantify human APOE in these samples. Figure 7A shows the size-exclusion chromatograms for all six individuals as tracked by TG content. In this column system, VLDL elutes between fractions 15 and 20, whereas LDL is centered at fraction 25 and HDL elutes between fractions 30–35. We again noted significant variations in VLDL TG among the subjects. The VLDL peak for each subject was collected, pooled, and subjected to LPL hydrolysis using the microplate method described previously. Like for cohort 1, Fig. 7B shows the ability of LPLs to hydrolyze the VLDL particles from the various individuals varied widely when compared on an equal TG basis. Each VLDL fraction was then assayed for APOB and APOE by ELISA. Figure 7C shows that LPL TG hydrolysis correlated negatively with the APOE/APOB ratio of the VLDL particles (*r*^2^ = 0.8887). These data confirm the results from cohort 1, supporting the concept that LPL activity is inversely related to the APOE content of human VLDL particles, regardless of the method of VLDL isolation (ultracentrifugation or size exclusion) or method of quantitating APOE (semiquantitative MS or ELISA).

**Fig. 8:**
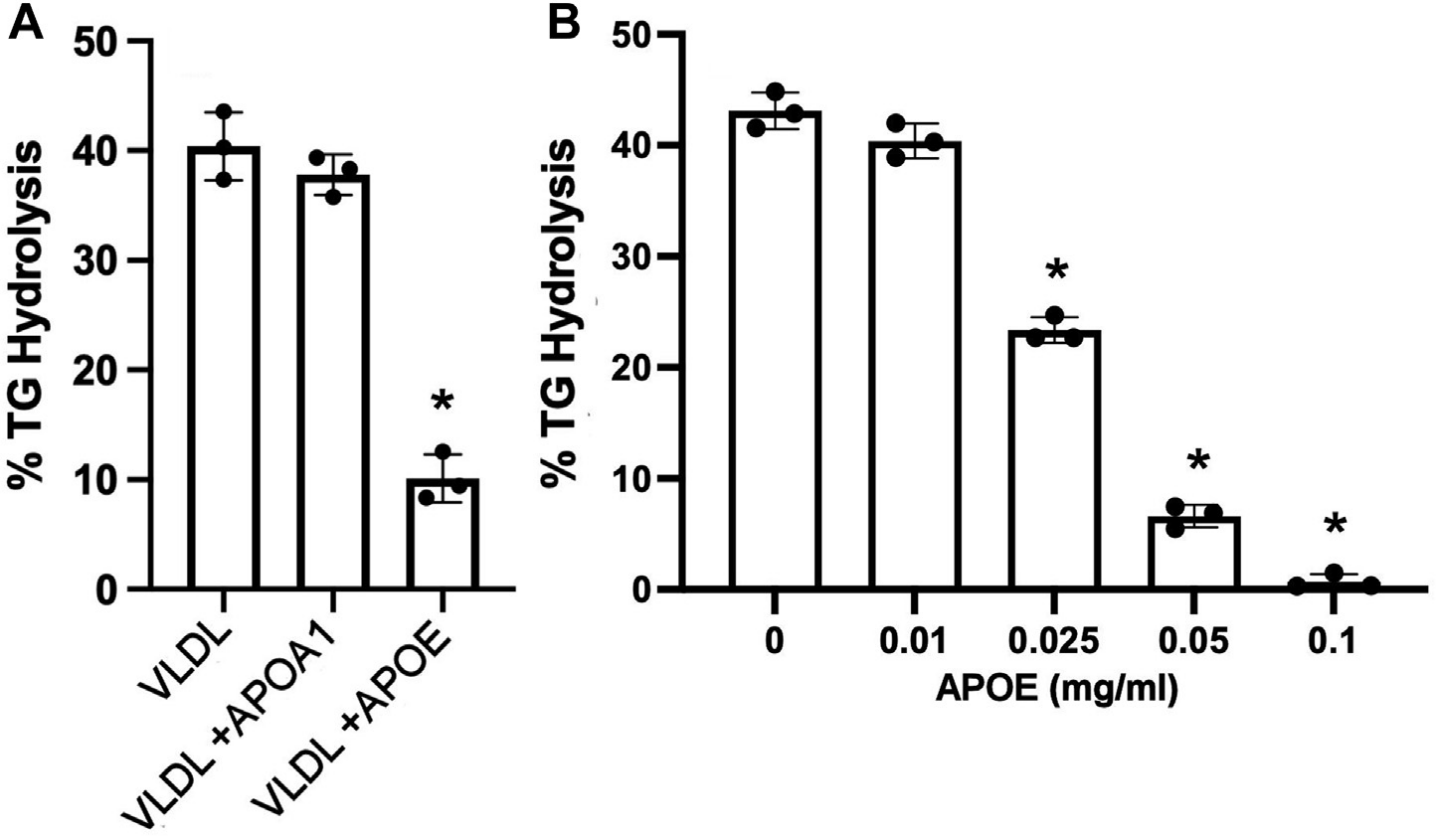
The effect of adding exogenous APOE on VLDL TG hydrolysis by LPL. A: VLDL (20 mg/dl of total protein) was incubated alone, with 0.05 mg/ml human plasma-purified APOA1, or with 0.05 mg/ml human plasma APOE for 1 h at 37°C prior to performing an LPL TG hydrolysis assay. A one-way ANOVA indicated difference exist among the samples (*P* < 0.001), and the ∗ shows a difference from VLDL only by Tukey test at *P* < 0.05. B: A similar experiment to that in panel (A) except that amount of human plasma APOE was systematically varied as shown. The same source of VLDL was used for each experiment, which were performed in triplicate with error bars representing one sample standard deviation. A one-way ANOVA indicated that differences exist among the samples (*P* < 0.001), and the ∗ shows a difference from VLDL with no APOE addition by Tukey test at *P* < 0.05.

To further evaluate the role of APOE in modulating LPL hydrolysis of VLDL particles, we performed an interventional experiment in which isolated human VLDL was incubated with lipid-free human plasma APOE or APOA1 (as a control) for 1 h at 37°C (Fig. 8A) prior to the LPL assay. Without apolipoprotein addition, LPL hydrolyzed the VLDL TGs effectively as in previous experiments. The same VLDL particles incubated with human plasma-purified APOA1 performed similarly. However, the addition of APOE protected VLDL TG from hydrolysis by LPL. This effect was concentration dependent (Fig. 8B). Incubating the VLDL particles (20 mg/dl) with 0.025 mg/ml of APOE (1.25 μg of APOE to 10 μg of VLDL) decreased TG hydrolysis by ∼40%, 0.050 mg/ml of APOE (2.5 μg of APOE to 10 μg of VLDL) decreased it by 80%, and 0.100 mg/ml (5 μg of APOE to 10 μg VLDL) protected the VLDL particles from LPL-mediated TG hydrolysis almost completely. We performed a similar experiment in which APOC2 was added back to VLDL in increasing concentrations. We noted variable effects with the two lowest additions of APOC2 having no effect on LPL activity, but we observed about a 10% increase in activity at 0.05 mg/ml of APOC2 followed by about a 20% decrease in activity at 0.10 mg/ml of APOC2 (supplemental Fig. S3).

**Fig. 9:**
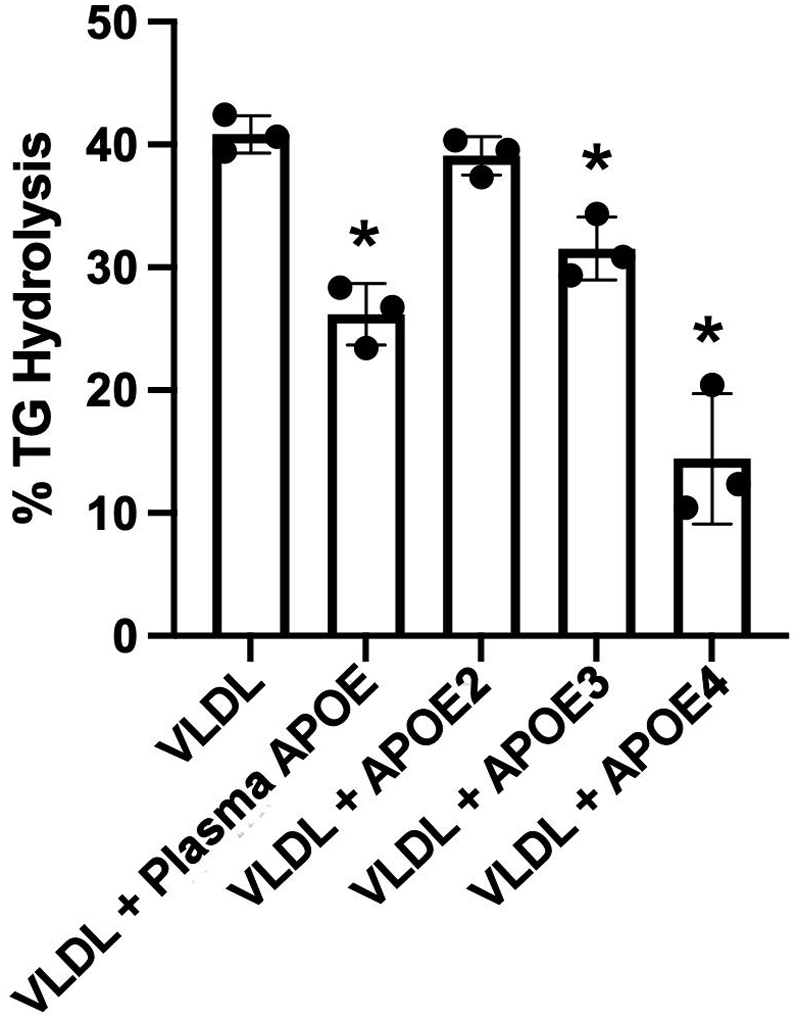
Effect of APOE isoforms on VLDL TG hydrolysis by LPL. The experiment was performed as in Fig. 8 except that 25 μg/ml of either human plasma APOE or recombinant APOE2, APOE3, or APOE4 were incubated with the VLDL particles (total protein of 20 mg/dl) for 1 h at 37°C prior to performing an LPL TG hydrolysis assay. The same source of VLDL was used for each experiment, which were performed in triplicate with error bars representing one sample standard deviation. *P* < 0.001 by one-way ANOVA. ∗ indicates difference from VLDL alone, *P* < 0.05 by Tukey test.

Human populations exhibit three major APOE isoforms that vary in amino acid substitution at positions 112 and 158 with profound effects on the lipid-binding and receptor-binding ability of the protein. Thus, we tested purified and recombinant APOE2, APOE3, and APOE4 in our system (Fig. 9). Again, VLDL alone was an effective substrate for human LPL. Addition of 25 μg/ml of human plasma APOE (which we presume is mostly APOE3) decreased TG hydrolysis as before. While pure APOE3 responded comparably to plasma APOE, addition of APOE2 did not significantly affect LPL-mediated TG hydrolysis versus no addition. Interestingly, APOE4 addition resulted in a robust reduction in percent of TG hydrolysis by LPL that superseded that of plasma APOE or APOE3.

## Discussion

LPL has been known to be a major player in the processing of TRL for nearly 70 years. However, because of its complex regulation by a number of factors present in both the target lipoproteins and anchoring points in the vessel wall, it has been difficult to derive a clear picture of how the apolipoprotein cargos of TRL impact LPL activity (33). In this study, we developed a plasma-like ex vivo assay that tests physiological VLDL particles isolated from human donors as substrates for soluble human LPL. Our results indicated that there is a wide variation in LPL activity against VLDL from different human subjects. This cannot be fully explained by the TG content of the particles. Proteomics analyses suggested that the level of APOE was the factor that best correlated (negatively) with hydrolysis by soluble LPL. This correlation was confirmed in a second cohort using orthogonal methods to isolate VLDL and quantify APOE. To our knowledge, this is the first study to apply modern proteomic techniques in natively isolated human VLDL particles in conjunction with a human LPL functional analysis under plasma mimic conditions.

The proteome of TRL is a critical factor in their rate of lipolysis by LPL. The classic example is APOC2, an activating cofactor for LPL (34). APOC2 may activate by directly binding LPL (35) or by affecting packing/surface pressure of the substrate lipid surface (23). Conversely, APOC3 has long been thought to inhibit LPL or at least disrupt APOC2 activation of LPL (20, 23). Our observation that APOC2 levels in VLDL did not strongly correlate with LPL activity is consistent with the idea that a relatively low threshold level of APOC2 is all that is required for efficient LPL activation, and additional copies per particle do not further activate, or even have detrimental effects (21), on LPL activity. Our experiments showing variable effects when APOC2 is added to VLDL is also consistent with this concept. A lack of negative correlation with APOC3 was perhaps more surprising as previous studies have shown that titrating APOC3 onto TRL progressively slowed LPL hydrolysis activity either in its free form or bound to GPIHBP1 (36). However, this was done with bovine LPL and not human as done here. The effects of APOC3 on LPL activity appear complex and may involve additional factors such as other APOCs and APOE (37). We look forward to exploring this in future studies.

APOE is well known to be key determinant in TRL uptake by remnant receptors and proteoglycans in the liver (38). It has also been shown to have complex effects on LPL activity in the circulation. Back when it was known as “arginine-rich polypeptide,” APOE was shown to attenuate the APOC2-mediated increase in LPL activity (39, 40). The idea that it worked along with other apolipoproteins was bolstered by the observation that addition of APOE to plasma inhibited LPL activity, but when added to clean TG emulsions, APOE actually enhanced activity (41). Rensen *et al.* (38) showed a concentration-dependent inhibitory effect of APOE in TG-rich emulsions injected into hepatectomized rats, an effect that could be ablated by selective modification of Arg residues in the protein. Similar effects were noted by Jong *et al.* (42) in liver perfusion of VLDL from APOE-deficient mice. However, other studies have produced contradictory results. For example, Yamada *et al.* (43) showed that purified human APOE added to human plasma significantly activated human LPL, whereas anti-APOE antibodies decreased activity. Zsigmond *et al.* (44) noted that TRL from APOE-deficient mice did not differ from those from WT mice as a substrate for exogenous human LPL. Finally, APOE was shown to nearly double the activity of the distinct but related hepatic lipase, whereas other apolipoproteins inhibited it (45).

Our results come down on the side of APOE playing a clear role in the modulation of LPL activity. A strength of our study was the isolation of human VLDL (physiological particles) by two different techniques and the use of MS and immunoassay to measure APOE for comparisons of LPL hydrolysis using the human enzyme. Our results were bolstered by follow-up experiments in which exogenous APOE was added back to physiological VLDL particles, confirming a concentration-dependent effect. Many previous studies used cross-species enzyme/substrates, synthetic emulsions that lack the proteomic/lipidomic complexity of native lipoproteins, or were performed in rodent models. While human and rodent lipolysis pathways appear generally similar in terms of regulation, the lipoprotein distributions differ dramatically (humans are “LDL” animals, whereas mice are “HDL” animals), and LPL tissue expression in mice differs from that of humans (46).

It is interesting to consider the potential mechanism by which APOE might alter LPL activity. We can envision three possible scenarios. First, the presence of APOE could alter the surface packing characteristics of the VLDL lipid surface, thus discouraging LPL binding or penetration into the particle to access core TGs. Second, APOE may directly bind to LPL and thus alter its activity perhaps through a conformational change. Third, APOE may attenuate the ability of other regulatory factors such as APOC2 or APOC3 to regulate LPL activity. While known to be surface active, APOE exhibits a similar collapse pressure at the air/water interface as APOA1 (which did not affect LPL activity in our study) and can be displaced from lipid surfaces by APOC3 (47). Also, the presence of APOE on microemulsions in the absence of other proteins does not inhibit LPL activity (41). These observations argue against the surface pressure modification idea. McConathy *et al.* (48) showed that a synthetic peptide corresponding to the receptor-binding domain of APOE was effective at inhibiting LPL activity. This is consistent with the results of Rensen (38) showing that modification of Arg residues (abundant in the receptor-binding domain) in APOE disrupted the LPL modulation activity. This would seem to favor the direct binding of APOE and LPL. However, this still does not explain why clean TG emulsions containing APOE alone fail to inhibit LPL. It seems that the more likely explanation is that the receptor-binding domain of APOE interacts with and modifies some other factor on TRL, which in turn affects LPL activity. Interestingly, recent work has shown that apolipoprotein A5 may affect LPL activity by interfering with the actions of ANGPTL3 and ANGPTL8 (49). APOE might impinge on this interaction as well. This is a strong argument for the use of physiological substrate lipoproteins containing proteomic diversity. We are currently investigating these hypotheses using our assay system.

Our results show that the APOE inhibition of LPL activity is isoform dependent with APOE4 being most effective, APOE2 not effective at all, and APOE3 being intermediate. The effectiveness of APOE4 may be explained by its high affinity for VLDL-sized particles. APOE4 differs from the common APOE3 by the substitution of an Arg residue for a Cys at position 112. This results in a conformational change that profoundly alters its lipid-binding affinity (50), favoring particles of low surface curvature like VLDL. APOE3 and E2, on the other hand, tend to prefer binding to particles with higher surface curvature like HDL. Thus, it is conceivable that a higher fraction of added APOE4 found its way to the substrate VLDL particles versus the other isoforms resulting in a larger inhibitory effect. The reason for the lack of effect of APOE2 versus APOE3 is less clear. The mutation that distinguishes it from APOE3, a Cys for Arg substitution at position 158, occurs near the binding site for the LDL receptor. Indeed, type III hyperlipidemia results from this mutation because these individuals cannot properly clear TRL remnants from the circulation. As discussed previously, previous studies have implicated the receptor-binding domain of APOE in the inhibition of LPL activity (48). Thus, it is reasonable to speculate that conformational changes around this domain are responsible for the lack of effectiveness of APOE2 in our assay. Unfortunately, our Institutional Review Board-approved protocol at the time of collection did not allow for tracking the APOE genotype in our small human cohorts. In the future, it will be interesting to study larger cohorts in which the APOE genotype can be tracked and correlated to LPL activity.

Finally, we were intrigued by the implications of our results for understanding TRL metabolism as a whole. APOE plays at least two apparently opposing roles with respect to TRL clearance from the plasma. On the one hand, its presence appears to slow LPL-mediated hydrolysis of VLDL TG—presumably favoring hyperlipidemia. On the other hand, APOE is a well-known ligand for the removal of TRL remnants in the liver (38)—thus working against hyperlipidemia. We speculate that the impact of these different roles may manifest depending on the location of the lipoprotein in the circulation. Perhaps APOE acts as a brake on lipolysis in tissues that may be sensitive to high concentrations of NEFAs and subsequent lipotoxicity. For example, excess NEFA in the pancreas can lead to pancreatitis, and prolonged exposure to NEFA can result in endothelial damage and vascular leakage. In the liver, on the other hand, the primary role of APOE is likely remnant particle uptake. In addition, the mix of TRL surface apolipoproteins is likely undergoing constant turnover in exchange with HDL. Perhaps, particular mixtures of apolipoproteins modulate the functions of APOE in different metabolic scenarios or locations. Further studies will be needed to work this out.

In conclusion, we have established that the content of APOE in VLDL has important implications on the LPL-mediated hydrolysis of cargo TGs on the particles. APOA2 may also play a role. Our current focus is aimed at understanding the mechanism behind these effects with the hope of developing interventional strategies that accelerate the turnover of TRL in the plasma compartment and limit their opportunities to accumulate in the vessel wall.

## Data availability

Proteomics data are available in the PeptideAtlas database. Dataset identifier: PASS01684.

## Supplemental data

This article contains supplemental data.

## Conflict of interest

The authors declare that they have no conflicts of interest with the contents of this article.
