# Supplementary material for: Apolipoprotein E content of VLDL limits LPL-mediated triglyceride hydrolysis
Source: J Lipid Res. 2021 Dec 1;63(1):100157. doi: 10.1016/j.jlr.2021.100157 (PMC8953696; doi:10.1016/j.jlr.2021.100157)
Supplement: Supplemental Figures S1–S3 [file mmc1.docx]

**Apolipoprotein E content of very low-density lipoprotein limits lipoprotein lipase-mediated triglyceride hydrolysis**

**ONLINE SUPPLEMENTAL DATA**

Brynne E Whitacre^1^, Philip Howles^1^, Scott Street^1^, Jamie Morris^1^, Debi Swertfeger^2^

and W. Sean Davidson^1^

^1^Dept. of Pathology & Laboratory Medicine, University of Cincinnati, Cincinnati, OH, USA

^2^ Department of Pediatrics, Cincinnati Children’s Hospital Medical Center, Cincinnati, OH 45229

**Supplemental Figure 1**


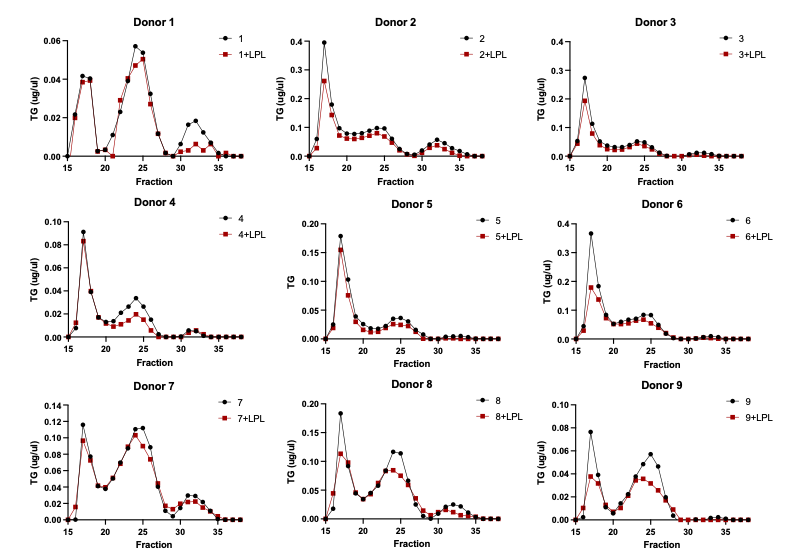


**Supplement Figure 1: Triglyceride profiles from size exclusion chromatography analysis of individual plasma donors pre and post addition of LPL.** 150μl of plasma from each donor in cohort 1 was separated by FPLC using a single Superose 6 column. Fractions were then analyzed for total triglyceride via colorimetric assay. Plasma was analyzed before (black trace) and after (red trace) incubation with human LPL as described in *Methods*.

**Supplemental Figure 2**

**Supplemental Figure 2: Comparison of the most abundant apolipoproteins on two representative donor’s VLDL particles one month apart.** 50 μg of total protein of VLDL isolated from each donor by ultracentrifugation was analyzed by mass spectrometry after delipidation, reduction, alkylation and digestion with trypsin as described in *Methods*. Panel (a) shows data from donor 1 and (b) shows data from donor 6. Run 1 was performed on VLDL isolated at time = 0 days and run 2 was performed on similarly isolated VLDL from the same donor at T = 30 days. Spectral counts for the most abundant proteins are shown. Representative data from samples run in triplicate. Note: Although VLDL samples were isolated one month apart, the mass spectrometry was performed on both samples at the same time.

**Supplemental Figure 3**

**Supplement 3: The effect of apoC-II on TG hydrolysis by LPL.** VLDL (20 mg/dL) was incubated alone or with different concentrations of human plasma APOC-2 for 1 h at 37 °C . Triglyceride concentration was quantified with and without the presence of LPL *in vitro.* A one-way ANOVA detected differences among the samples at p<0.001 and the stars represent differences from the VLDL with no added apolipoprotein at p<0.05 by a Tukey test.
